# Use of Confocal Laser as Light Source Reveals Stomata-Autonomous Function

**DOI:** 10.1371/journal.pone.0000036

**Published:** 2006-12-20

**Authors:** Roberto C. Cañamero, Hernán Boccalandro, Jorge Casal, Laura Serna

**Affiliations:** 1 Environmental Sciences Faculty, University of Castilla-La Mancha Toledo, Spain; 2 Instituto de Investigaciones Fisiológicas y Ecológicas Vinculadas a la Agricultura (IFEVA), Faculty of Agronomy, University of Buenos Aires Buenos Aires, Argentina; 3 Consejo Nacional de Investigaciones Científicas y Técnicas Buenos Aires, Argentina; Purdue University, United States of America

## Abstract

In most terrestrial plants, stomata open during the day to maximize the update of CO_2_ for photosynthesis, but they close at night to minimize water loss. Blue light, among several environmental factors, controls this process. Stomata response to diverse stimuli seems to be dictated by the behaviour of neighbour stomata creating leaf areas of coordinated response. Here individual stomata of *Arabidopsis* leaves were illuminated with a short blue-light pulse by focusing a confocal argon laser. Beautifully, the illuminated stomata open their pores, whereas their dark-adapted neighbours unexpectedly experience no change. This induction of individual stomata opening by low fluence rates of blue light was disrupted in the *phototropin1 phototropin2* (*phot1 phot2*) double mutant, which exhibits insensitivity of stomatal movements in blue-illuminated epidermal strips. The irradiation of all epidermal cells making direct contact with a given stoma in both wild type and *phot1 phot2* plants does not trigger its movement. These results unravel the stoma autonomous function in the blue light response and illuminate the implication of PHOT1 and/or PHOT2 in such response. The micro spatial heterogeneity that solar blue light suffers in partially shaded leaves under natural conditions highlights the physiological significance of the autonomous stomatal behaviour.

## Introduction

Stomatal pores are located on the plant epidermis and regulate CO_2_ uptake for photosynthesis and water loss to drive transpiration. Stomatal opening is induced by several environmental factors, among them, blue light [1]–[3]. When either entire plants or epidermal strips adapted to darkness are exposed to low fluence rates of blue light, the stomata open their pores [4]–[6]. The blue-light receptors PHOTOTROPIN1 (PHOT1) and (PHOTOTROPIN2) PHOT2 control this response. In a redundant fashion, they mediate the stomatal opening, with both *phot1* and *phot2* single mutants being indistinguishable from wild-type plants when epidermal strips are illuminated with blue light, and *phot1 phot2* double mutant exhibiting insensitivity of stomatal opening under these conditions [4]. Both, PHOT1 and PHOT2 are composed by a serine/threonine kinase domain located within the carboxy-terminus and repeated photosensory motifs referred LOV1 and LOV2 in the amino-terminus [7], [8]. Blue-light-specific stomatal opening has an action spectrum typical of other blue-light responses of plants, showing a maximum at 450-nm and two minor peaks at 420-nm and 470-nm [9], which closely matches the absorption spectra of the LOV domains of PHOT1 and PHOT2 [9]–[12].

In dark-grown seedlings, both co-sedimentation experiments with plasma membrane enzymes [13], [14] and aqueous two-phase partitioning [15]–[18] place PHOT1 at the plasma membrane. In addition, experiments with right-side-out plasma membrane vesicles show that it is associated only with the inner surface of the plasma membrane [16]. More recently, brief light treatments in *PHOT1-GFP* etiolated seedling have shown that a fraction of PHOT1 is released from the cell membrane to the cytoplasm in response to blue light [19]. Analysis of *PHOT2-GFP* has just shown that in the dark PHOT2 localizes, like PHOT1, mainly to the plasma membrane [20]. However, blue light illumination induces its association with the Golgi apparatus [20].

Stomata response to diverse stimuli seems to be dictated by the behaviour of neighbour stomata creating leaf areas of coordinated response [21], [22]. For example, when a single stoma is exposed to a current of dry air, adjacent stomata also tend to close, despite being not exposed to the signal [21], [23]. This coordinated behaviour is apparently due to hydraulic coupling among stomata [21], [24]. Here, we experimentally address whether stomata might function autonomously in the blue light response by individuals cells irradiation with a laser. We show that stomata act independently regardless of the behaviour of their neighbours and highlight the implication of PHOT1 and/or PHOT2 in such response. The physiological advantage of the stomatal autonomous function is discussed.

## Results/Discussion

**Figure 1:**
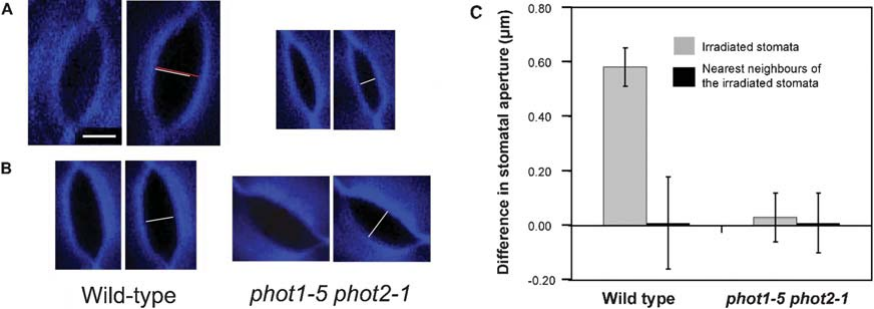
Stoma autonomy in its blue light-induced opening Individual stomata were irradiated with a short pulse of blue light by focusing an argon laser attached to a confocal microscope. (A) Irradiated stomata. (B) Nearest neighbour of the irradiated stoma. Confocal sections showing the stomatal opening in both wild type and *phot1-5 phot2-1* double mutant, before (left) and two hours later (right) the blue-light treatment. (C) Differences between the stomatal opening before and two hours after the blue light treatment in both irradiated stomata and their nearest neighbours dark-adapted stomata. Wild-type irradiated stomata increased pore opening. In contrast, the irradiated stomata of the *phot1-5 phot2-1* double mutant experienced no change. The nearest neighbours to the irradiated stomata remained unaltered in their movements. Bars indicate the mean of at least 15 measurements with standard deviations. Calcofluor staining (0.1%) produced a blue fluorescence in all cell walls when excited with a 405-nm laser line of a diode laser. White line shows the initial opening; red one represents the final aperture. Scale bar: 3 µm; all images are the same magnification.

We used simultaneously both a 458-nm line and a 476-nm line of an argon laser attached to a DMIRB inverted Leica TCS SP2 confocal microscope, to investigate whether stomata might function autonomously in response to blue light. Individual stomata were exposed to 10 µmol m^−2^ s^−1^ of blue-light for 10 s, and their neighbours were maintained in the dark (or illuminated with a 405-nm laser line of the diode laser, not shown). Of a total of 20 illuminated stomata, 17 (85%) increased the size of their pores (Figure 1A and 1C), whereas their dark-adapted neighbour exhibited no change (100%; *n* = 15; Figure 1B and 1C). This resulted in rejecting the null hypothesis of independence between the blue-light irradiation and the number of opened stomata (P<0.0001). This result unravels the stomatal-autonomous opening in the blue light response, and also it demonstrates that the signal that triggers stomatal movements does not transmit across the epidermal tissue, at least from stoma to stoma. The 10 s-pulse illumination of 10 µmol m^−2^ s^−1^ resulted in an increase in aperture (Figure 1C), which does not differ from the data obtained when epidermal strips of Arabidopsis were illuminated with continuous 5 µmol m^−2^ s^−1^ blue light [5]. It should be noted that, like other authors discussed [12], [25], the very low fluencies employed in our experiments ensure that the blue-light-induced stomatal movements are due to the photoreceptors rather than to photosynthesis.

**Figure 2:**
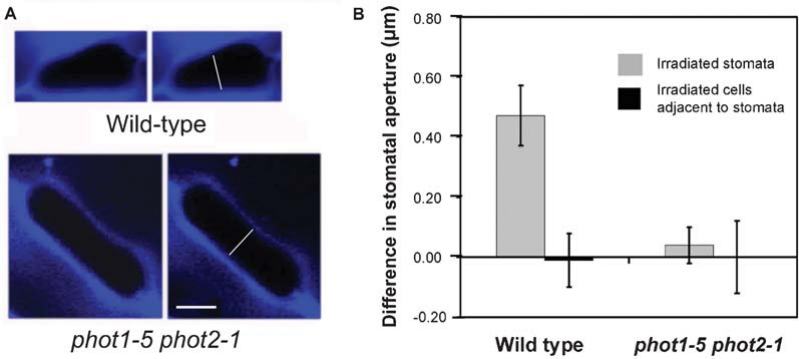
Blue light-induced stomatal movement signals are not transmitted from non-stomatal epidermal cells to their adjacent stomata A short pulse of blue light from the argon laser was applied to all epidermal cells adjacent to randomly selected stomata in both wild type and *phot1-5 phot2-1* double mutant. Such irradiation caused no effect in the stomatal movements. (A) Confocal sections show the stomatal apertures before (left) and two hours later (right) the non-stomatal epidermal cells illumination. (B) Differences between the stomatal aperture before and after the blue light treatment. Note that illuminated stomata increased opening in the wild type, and remained unaltered in the *phot1-5 phot2-1* double mutant. Bars show the mean of at least 15 measurements with standard deviations. Images show staining with calcofluor resulting in strong fluorescence in cell walls. Scale bar: 3 µm; all images are the same magnification.

In supporting the absence of a blue-light induced cell signalling across the epidermal tissue, irradiation of stoma neighbour epidermal cells with a short-pulse of blue light caused no effect on the opening of the adjacent stoma. The three neighbour epidermal cells that surround a given stoma were simultaneously illuminated, but the stoma making contact with such cells remained unaltered (Figure 2A). A total of 19 stomata (100%; *n* = 19) exhibited this behaviour (Figure 2B). Control stomata in these peels were illuminated, and as it might be expected they increased pore opening (*n* = 19; 90.5%; Figure 2B). This resulted in rejecting the null hypothesis of independence between the cell type that is irradiated and the number of opened stomata (P<0.0001).

The induction of stomatal opening in wild type plants by blue light was disrupted in the *phot1 phot2* double mutant (Figure 1A and 1C; P<0.0001). Of a total of 15 illuminated stomata, every one of them showed insensitivity of stomatal opening to the blue light-pulse. This result supports the previously established function of both PHOT1 and PHOT2 in the stomatal aperture in the blue-light response [4], and it demonstrates the cell-autonomous roles for these genes in controlling the stomatal movements. Like in the wild type, the dark-adapted neighbours of the illuminated stomata experienced no change (*n* = 20; 100%; Figure 1B and 1C). The three non-stomatal epidermal cells that make contact with every stoma were also illuminated and like in the wild type, the adjacent stomata did not increase the size of their pores (*n* = 15; 100%; Figure 2A and 2B). These results support the absence of a signalling response to blue light across the epidermal tissue. Stomata, in the peels of *phot1 phot2* where epidermal cells adjacent to stomata were irradiated, were also illuminated and they experienced no change in their movements (*n* = 19; 100%; Figure 2B; P<0.0001, compared with wild type plants).

**Figure 3:**
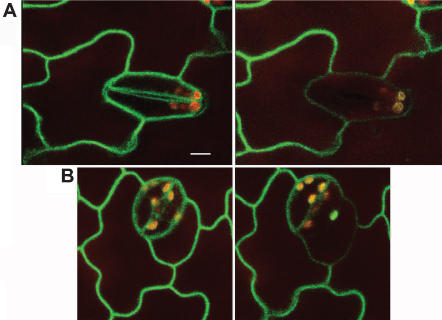
Dissociation of PHOT1 from the plasma membrane in blue-light irradiated cells (A) Stomata blue-light irradiation. Confocal section of the epidermis of a dark-adapted *PHOT1-GFP* seedling showing all GFP signal attached to the cell surface (left). Section of the same field 3 min after the initial scan (right). The intensity of the GFP signal decreased in the irradiated stoma. No changes were detected in its neighbour epidermal cells. (B) Stomatal neighbour cell irradiation. Epidermal sections before (left) and after (right) the blue-light illumination. The argon laser provided the blue-light source. Scale bar: 3 µm; all images are the same magnification.

When paired guard cells of dark adapted *PHOT1-GFP* seedlings were irradiated with a short blue-light pulse, the intensity of the GFP signal at the cell surface decreases in the irradiated cells (Figure 3A). Similarly, when stoma neighbour epidermal cells were illuminated, the cell surface GFP signal decreased specifically in such cells (Figure 3B). The blue-light treatment also affected the common fraction of the irradiated neighbour cell(s). This loss of the GFP intensity in the cell surface of the irradiated cells supports the idea that PHOT1 is released from the cell membrane to the cytoplasm in blue-light irradiated seedlings [19], and evidences on the cell autonomy of such process. The changes in the guard cells GFP intensity were evident three minutes after the scan. However, no change in the stomatal aperture was detected at this time, indicating that PHOT1 diffusion does not imply an immediate stomatal opening. In addition, the absence of stomatal movement when the laser was applied to all epidermal cells surrounding a given stoma together with the PHOT1 guard cell diffusion from the common fraction of the irradiated epidermal cell, indicate that the blue-light induced change restricted to the cell membrane is not sufficient to induce stomatal opening. This suggests that, in addition to plasma-membrane processes, stomatal opening requires changes directly induced by blue-light in the cytoplasm of guard cells.

A recent report has demonstrated that cryptochromes (CRY1 and CRY2) act additively with phototropins (PHOT1 and PHOT2) to mediate blue light-mediated stomatal opening [6]. The lack of a residual response to blue light in the *phot1 phot2* double mutant in our experiments compared to others where epidermal strips were exposed to prolonged blue plus red light [6] suggests that cryptochrome action might require a longer blue-light exposure, require a red background and/or function in a non-cell-autonomous manner.

In summary, we have demonstrated that stomata blue-light illumination is both sufficient and necessary to mediate stomatal opening, which, in addition, depends on PHOT1 and/or PHOT2 activity. This scenario is consistent with the observation that onion guard cells protoplasts swell when illuminated with blue light, but non-stomatal epidermal cell protoplasts do not swell under the same conditions [26]. The stomatal autonomy seems to extend in response to abscisic acid. Certainly, when single guard cells are injected with cyclic ADP-ribose, which mediates the abscisic acid-induced stomatal closure, its turgor decreases while those from the uninjected partner remains unchanged [22], [27]. However, our finding that stomata act independently of the behaviour of those around them, contrasts with recent works suggesting that stomatal function is dictated by that of neighbour stomata [10], [23], [24], and opens the question on why the blue-light pulse has not a similar effect in the stomatal behaviour.

**Figure 4:**
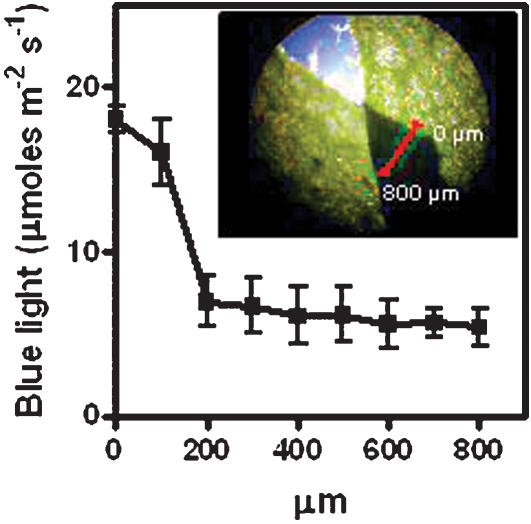
Nearby parts of an Arabidopsis leaf can experience steep differences in irradiance Solar blue light decreases sharply between the unshaded (0 µm) and shaded portions of the abaxial side of an Arabidopsis leaf. The average distance among stomata in Arabidopsis varies between 50 and 100 µm, indicating that neighbour stomata may be exposed to widely different light conditions. Data are means and standard errors of three replicates. Inset, Image of overlapping leaves illustrating the sharp transition between illuminated and shaded areas.

But, what advantage might stomata-autonomous function induced by blue-light confer on the plant? When a leaf is partially shaded by another leaf, incident blue-light irradiance is below the saturation value of phototropin action in the shaded region and above saturation in the lighted area (Figure 4). In addition, such change in blue-light irradiance occurs in a micrometric distance (Figure 4), similar to the average distance between two neighbour stomata [28]. In this context, the stomata-autonomous function would allow the opening of the lighted stoma, while maintaining the shaded neighbour one in a relatively closed state. This stomata-autonomous behaviour would optimise the balance between water loss and CO_2_ acquisition.

Light regulates many developmental and physiological processes in both plant and animal systems. Blue-light, for example, triggers de-etiolation, phototropic curvature, chloroplast movement, and stomatal opening [2], [29], [30]. The possibility of using the laser of a confocal microscope as a light source, opens an exciting and long way to investigate the cellular autonomy and/or cell-to-cell signalling in these and many others light-induced process.

## Materials and Methods

### Plant materials and growth conditions

The blue-light dependent stomatal movements of the double mutant *phot1-5 phot2-1* and its corresponding wild-type strain (Columbia) were previously described [4], [6], [31]. The *phot1-5* mutant (originally *nph1*) is a null allele [7]. The *phot2-1* mutant (originally *npl1*) results from a stop codon between the LOV2 and kinase domain, so active PHOT2 protein is not produced in this genetic background [4]. Transgenic plants expressing the *PHOT1-GFP* gene under the control of the *PHOT1* promoter have been described [19]. Seedlings were grown on soil at 20°C with 16 h of light/8 h of dark under fluorescent lamp (100 µmol m^−2^ s^−1^). The relative humidity in the growth chambers was maintained at 60%.

### Stomatal aperture measurements

Abaxial peels of mature, fully expanded leaves (3- to 6-week-old plants), were detached in the early morning and kept in the dark for 1 h under an incubation solution containing 0.1 mM CaCl_2_ and 20 mM KCl. Dark-adapted peels were mounted on slides under a drop of the incubation solution. Apertures of randomly selected stomata were measured from transmission images taken at 10.000-fold magnification. They, or their three neighbour epidermal cells, were illuminated with blue light for 10 seconds. Two hours later, transmission images were again monitorized to measure the final stomatal apertures. Controls were included in every set of laser irradiations. At least, 15 stomata, from a total of at least 8 leaves, were monitorized for each treatment and genetic background. Results were evaluated by using χ2-test (99% confidence level).

### Blue-light treatment

Transmission images from both dark-adapted and illuminated seedlings were monitorized with a DMIRB inverted Leica TCS SP2 confocal microscope using a 405- nm laser line of a diode laser and excitation beam splitter substrat. The scan speed was 400 Hz and the pinhole size of 1 Airy unit. Images acquisition was performed by using a 63x/NA 1.40-0.60 PL APO (oil immersion) objective with an 8× zoom. Images were recorded with 512×512 pixels and arranged using Adobe Photoshop 6.0.

The blue-light treatment was performed by irradiating the cells simultaneously with both a 458-nm laser line and 476-nm laser line of an argon laser, excitation beam splitter RSP 500, beam expander 1, and the confocal pinhole size of 1 Airy unit. A single plane of the sample was irradiated for 10 s at 10 µmol m^−2^ by using 16 line average single scan and 512×512 pixel format with both lines at full power, and the argon laser level around 25%. Three regions of interest (ROI's), selected by hand-drawing (Poly tool) the cellular contour in a sample Z-section of the three cells that surround to a given stoma, were used to irradiate simultaneously such cells. Because stomata have an ellipsoidal shape, simple ROI- ellipsoidal scanning (ellipse tool) was used to illuminate simultaneously the two guard cells. Control stomata (referred as dark-adapted neighbours) were irradiated by setting both the 458-nm laser line and 476-nm laser line at 0% of available power.

### Light transition measurements

Blue light (400–500 nm) was measured with a fibre optics touching the abaxial face of an Arabidopsis leaf. The fiber optics was connected to a spectroradiometer (Analytical Spectral Devices Field Spec Pro FR) and positioned at 100 µm intervals with a calliper. The partial shade was produced by another Arabidopsis leaf placed 1 cm above the measured leaf. The leaf photograph was taken with a digital camera connected to an optic microscope. All data were collected at sunny middays.
